# MiR-302a/b/c/d cooperatively sensitizes breast cancer cells to adriamycin via suppressing P-glycoprotein(P-gp) by targeting MAP/ERK kinase kinase 1 (MEKK1)

**DOI:** 10.1186/s13046-016-0300-8

**Published:** 2016-02-03

**Authors:** Lin Zhao, Yan Wang, Longyang Jiang, Miao He, Xuefeng Bai, Lifeng Yu, Minjie Wei

**Affiliations:** Department of Pharmacology, School of Pharmacy, China Medical University, No.77 Puhe Road, Shenyang North New Area, Shenyang City, 110122 Liaoning China

**Keywords:** MiR-302, Breast cancer, MEKK1, Drug resistance

## Abstract

**Background:**

The importance of individual microRNAs (miRNAs) in tumor has been established in different cancers. However, their association with tumor chemoresistance has not been fully understood. Previously, we found two novel MDR-associated microRNAs (miRNAs). In this report, we investigated the combined effects of miRNA gene cluster in chemoresistance of breast cancer.

**Methods:**

This study was performed in two different breast cancer cell lines (MCF-7 and MCF-7/ADR). The levels of miRNAs and mRNA expression were determined by using Quantitative Real-Time PCR. Western blotting was used to detect the levels of protein molecules. Cell viability was assessed by MTS assay. Bioinformatics and Luciferase reporter assay was performed to examine miRNA binding to the 3′-UTR of target genes.

**Results:**

The miR-302S family including miR-302a, miR-302b, miR-302c, and miR-302d was significantly down-regulated in P-glycoprotein (P-gp)-overexpressing MCF-7/ADR cells. Overexpression of miR-302 increased intracellular accumulation of ADR and sensitized breast cancer cells to ADR. Most importantly, miR-302S produced stronger effects than each individual member alone. The four miRNAs cooperatively downregulate P-gp expression in regulating drug sensitivity. However, our results showed that the suppression of P-gp expression by miR-302 is not through typical miRNA-mediated mRNA degradation but at the level of protein and transcription. Further studies identified MAP/ERK kinase kinase 1 (MEKK1) as a direct and functional target of miR-302. miR-302 showed combinatorial effects on MKEE1 repression and MEKK1-mediated ERK pathway. The suppression of P-gp by miR-302 was reversed by MEKK1 overexpression.

**Conclusion:**

Our results indicate that miR-302 cooperatively sensitizes breast cancer cells to adriamycin via suppressing P-glycoprotein by targeting MEKK1 of ERK pathway. miR-302 gene cluster may be a potential target for reversing P-gp-mediated chemoresistance in breast cancer.

## Background

Breast cancer is the most common malignant tumor in women [1]. Despite advances in diagnosis and treatment approaches, the mortality due to breast cancer still remains very high. This is attributable to the fact that cancer cells are able to develop mechanisms of resistance to the therapeutic treatment, a process known as chemoresistance, which continues to be a major clinical obstacle to the successful treatment of cancer [2, 3]. It may be caused by drug efflux by transporters, inactivation by detoxification enzymes, the altered expression of pro-antiapoptotic proteins, changes in tumor suppressor gene expression, or the increased activity of DNA repair mechanisms [4–6]. Recent evidence has demonstrated that microRNAs (miRNAs) take part in these processes.

miRNAs is a type of endogenous non-coding RNA (ncRNA). They modulate protein expression by promoting RNA degradation and inhibiting transcription after binding to the 3′-untranslated region (3′-UTR) of mRNA [7]. Accumulating evidence has demonstrated that miRNAs are responsible for post-transcriptional regulation and participate in nearly all biological processes [8]. miRNAs play an important role in tumorigenesis, with some able to act as oncogenes, others as tumor suppressors and others displaying either oncogenic or tumor-suppressive activities, depending on the tissue and tumor context [9, 10]. They have recently emerged as diagnostic and prognostic markers for successful therapeutic responses. Recent investigations have revealed that miRNAs are involved in the development of drug resistance, for example, miR-520 h directly affects cell chemosensitivity in breast cancer cells [11]. miR-130a plays a role in chemosensitivity in prostate [12] and ovarian cancer [13], and the expression of miR-124 is associated with chemosensitivity in tongue squamous cell carcinoma(TSCC) patients [14].

Bearing in mind that the 3′ UTR of a single gene is frequently targeted by several different miRNAs, and that any one given miRNA can have several targets, some belonging to the same functional network or signaling pathway. A coordinated action of miRNAs on their targets was proposed. For example, for the known targets of miR-375, a combined addition of miR-124 and let-7b led to a synergy in target inhibition [15]. Similarly, the expression of miR-16, miR-34a and miR-106b altered the cell cycle. Combining these miRNAs resulted in cell cycle arrest that was stronger than for each of the miRNAs alone [16]. The regulation of the tumor suppressor FUS1 in cancer cells depends on the presence of at least three miRNAs (miR-93, miR-98,miR-197 and additional unidentified miRNAs) [17]. To date, the specific miRNAs involved in the cooperatively regulating drug resistance have yet to be characterized. In our previous study, we performed miRNA microarray analysis to investigate the differential miRNA expression profiling in parental MCF-7 cells and its derivative mitoxantrone(MX)-resistant MCF-7/MX cells. We found that miR-302S family including miR-302a, miR-302b, miR-302c, and miR-302d was significantly downregulated in MCF-7/MX cells. We also found that miR-302 inhibit BCRP-mediated MX resistance *in vitro* and *in vivo* study [18]. However, the involvement of miR-302S in the development of other drug resistance usually used in breast cancer therapy is unclear.

In this study, a multiple-drug resistant cell line, MCF-7/Adriamycin (ADR), was derived from MCF-7 cells by exposing them to gradually increasing concentrations of adriamycin (ADR). We experimentally demonstrated that miR-302 cluster, including miR-302a, miR-302b, miR-302c and miR-302d exert a combinatorial effect on the reverse the drug resistance of breast cancer cells. The four miRNAs cooperatively downregulate P-glycoprotein (P-gp)expression in regulating drug sensitivity. However, our results showed that the suppression of P-gp expression by miR-302a, miR-302b, miR-302c and miR-302d is not through typical miRNA-mediated mRNA degradation but at the level of protein and transcription. Further, we demonstrate that the four miRNAs directly bind to and down-regulate levels of MAP/ERK kinase kinase 1 (MEKK1), a member of the MAPK Kinase (MAP2K) Kinase (MAP3K) family. In cancer cells, miR-302S showed combinatorial effects on MEKK1 repression and MEKK1-mediated ERK pathway. Importantly, the suppression of P-gp by miR-302 was reversed by MEKK1 overexpression, suggesting that miR-302 cooperatively sensitizes breast cancer cells to adriamycin via suppressing P-gp by targeting MEKK1 of ERK pathway. Altogether, our results identify four specific miRNAs that regulate drug sensitivity and indicated the combination of miRNAs is required as an effective therapeutic strategy, and further elucidated the functional significance of the four miRNA combination.

## Methods

### Cell lines

The human breast cancer MCF-7 cell line was purchased from the American Type Culture Collection. The adriamycin (ADR)-resistant MCF-7 cells (MCF-7/ADR) were derived from the human breast cancer cell line MCF-7, was maintained in the presence of 1 μM adriamycin. A series of MCF-7 cells with incremental resistance to adriamycin were established by doxorubicin challenge at the starting concentration of 1 nM. After cells were tolerable, a double concentration of adriamycin was applied. The process was repeatedly performed to increase cell tolerance to adriamycin. The resulting 12^th^ generations, MCF-7/ADR were cultured in DMEM (Invitrogen, Carlsbad, CA) supplemented with 10 % FCS and were added with the indicated adriamycin concentrations for resistance maintenance.

### Cell transfection

MCF-7 and MCF-7/ADR cells were transfected with 20 nM miR-302a, miR-302b, miR-302c, miR-302d, and miR-302S (miR-302a-d) mimics or negative control miRNAs (NC) using Lipofectamine 2000, according to the manufacturer’s instruction. After transfection for 48 h, cells were used for Western blot and qRT-PCR.

### MTS assay for proliferation activity

MCF-7 and MCF-7/ADR cells were seeded onto 96-well plates at a density of 1,000 cells/well. After culture for 24 h, cells were transfected with 20 nM miR-302 mimics for 24 h. Then, cells were treated with serial dilutions of drugs for 48 h, followed by treatment with MTS (5 mg/ml, Promega, WI, USA) for 2 h. The absorbance at 490 nm was measured using a multi-mode reader (LD942, Beijing, China). The IC_50_ (50 % inhibitory concentration) value, which represents the concentration of the drug that demonstrates 50 % of cell growth inhibition, was calculated by normal probability transforms according to the relationship of drug concentration and inhibition rate. The probit regression models of SPSS 16.0 software were used for computation.

### Quantitative reverse transcription-PCR

Quantitative reverse transcription-PCR (qRT-PCR) was performed to detect the relative expression of mRNA. Briefly, total RNA was isolated from MCF-7 or MCF-7/ADR cells using Trizol reagents (Invitrogen, CA, USA) according to the manufacture’s protocol. RNA was reverse transcribed into complementary DNA using M-MLV reverse transcriptase (Promega, WI, USA). RT-PCR was performed using SYBR Premix Ex Taq™ II kit (Takara, Dalian, China) in a final volume of 10 μl mixture containing 1 μl cDNA, 0.5 μl of each primer, and 5 μl SYBRGreen. The reaction condition was as follows: 37 °C, 15 min, 85 °C, 5 s; 95 °C 5 min, followed by 95 °C, 10 s, 56 °C, 45 s and 72 °C, 20 s for 45 cycles of amplification. Relative mRNA expression was normalized to GAPDH expression.

### Luciferase report gene detection

The human P-gp 3′-UTR (380 bp) or MEKK1 3′-UTR (2472 bp) was cloned into the XbaI site of the pGL3 vector (Promega, WI, USA). Mutations in the miRNA-binding site were generated using RCR-based mutagenesis (Takara, Dalian, China). Luciferase reporter vectors (control), or vectors containing wild type (pGL3- P-gp -3′UTR-Full or pGL3- MEKK1 -3′UTR-Full) or mutated (pGL3-MEKK1-3′UTR-Mut) 3′-UTRs of MEKK1 mRNA were co-transfected into HEK-293T cells with miR-302a, miR-302b, miR-302c, miR-302d, or miR-302S mimics, using Lipofectamine 2000. After 24 h, luciferase activity was detected using the Dual Luciferase Reporter Gene Assay kit (Promega, WI, USA). Relative luciferase activity normalized to the negative control was used for comparison among groups.

### Western blotting

MCF-7 or MCF-7/ADR cells were washed with ice-cold PBS for 2 times, added RIPA lysis buffer, lysed on ice for 30 min, scrapped off, transferred into EP tube and centrifuged at 4 °C, 12,000 × g for 30 min. The supernatants were collected and protein concentration was determined using BCA protein quantitation kit. Each sample with 60 μg protein was added 1× SDS sample buffer [100 mmol/L Tri-HCl (pH 6.8), 4 % SDS, 0.2 % bromophenol blue, 20 % glycerol, 200 mmol/L β-mercaptoethanol], and denatured at 95 °C for 5 min. Proteins were separated by 10 % SDS-PAGE electrophoresis and transferred to 0.45 μm NC membrane. Membrane was blocked with 5 % skim milk for 1 h, and incubated with monoclonal antibodies (1:1,000 to 1:2,000 dilution) at 4 °C overnight. The membrane was then incubated with horseradish peroxidase-linked goat anti-rabbit secondary antibodies (Santa cruz, CA, USA) at room temperature for 1 h, washed with PBST three times, and detected with a chemiluminescent detecting system (Amersham, Freiburg, Germany).

### ADR accumulation assay

ADR accumulation assay was performed as described previously [19] with modifications. Briefly, cells were exposed with 5 μM ADR for 2 h at 37 °C in the darkness. The ADR accumulation was stopped by addition of ice-cold phosphate-buffered saline (PBS). The intracellular ADR level was determined by measuring ADR fluorescence using a flow cytometer (Becton–Dickinson).

### RNA degradation analysis

48 h after MCF-7/ADR cells were transfected with 20 nM miR-302 mimics, actinomycin D (Sigma, CA, USA) was added to a final concentration of 5 mg/mL to block de novo RNA synthesis. Cells were harvested at 0, 2, 4, 6, and 8 h following actinomycin D treatment. P-gp or MEKK1 mRNA levels were determined by qRT-PCR, and normalized to GAPDH mRNA levels. All treatments were conducted in triplicate.

### Statistical analysis

Data were analyzed using the SPSS statistics 16.0 software package. Results are presented as mean ± standard deviation (SD). One-way ANOVA was used to compare differences among groups, followed by LSD post-hoc tests. The *P* values < 0.05 were considered statistically significant.

## Results

### miR-302a/b/c/d is downregulated in MCF-7/ADR cells overexpressing P-gp

**Fig. 1:**
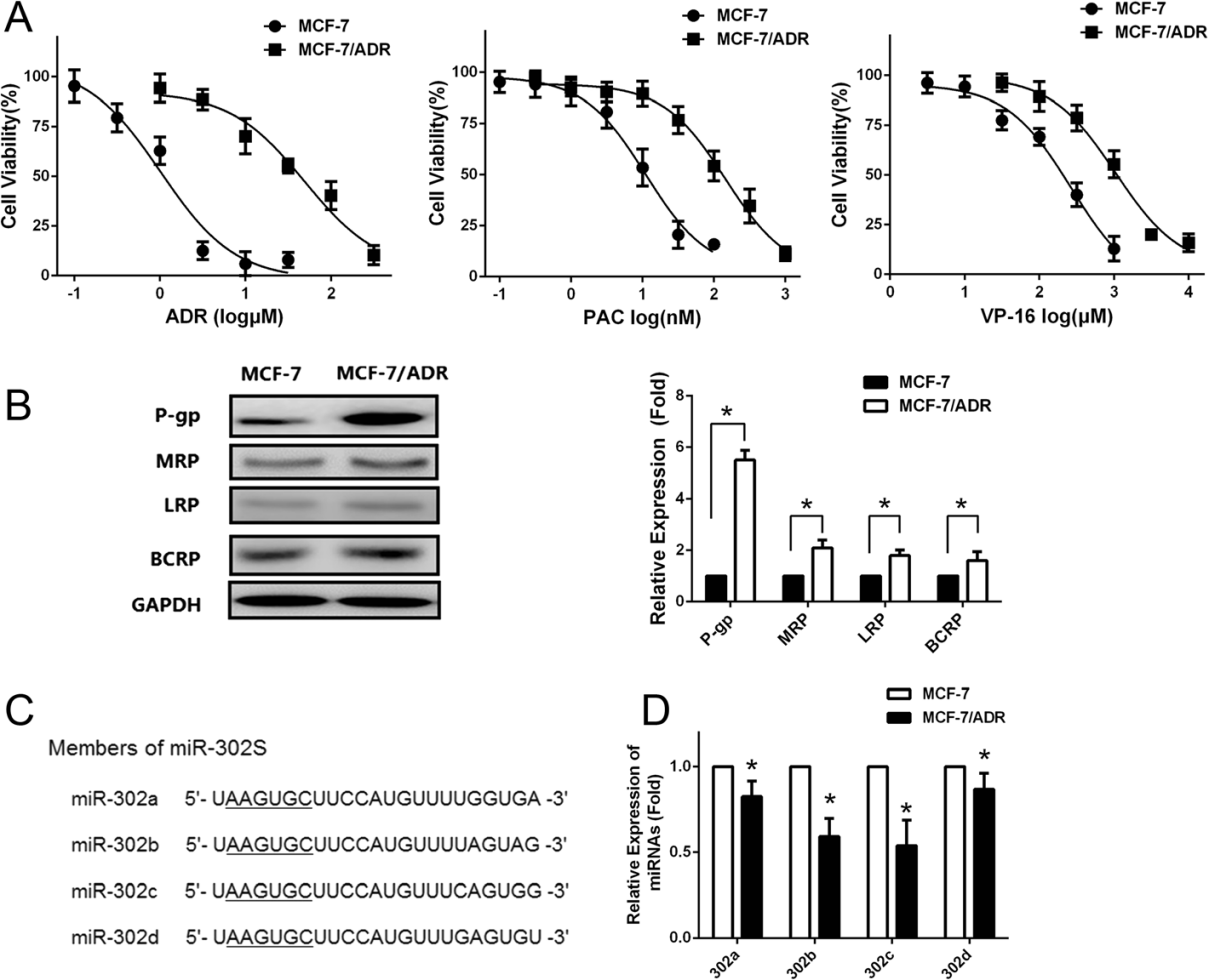
Downregulation of miR-302 expressions in MCF-7/ADR cells. **a** Cells were treated with various concentrations of ADR, PAC or VP-16. Survived cells were measured by MTS assay. MTS assay shows that MCF-7/ADR cells are much more resistant to ADR, PAC and VP-16 than MCF-7 cells (**b**) Left: Western blotting analysis showing the protein expression of MRP, P-gp, LRP, and BCRP in MCF-7 and MCF-7/ADR cells. GAPDH was used as an internal loading control. Right: Densitometric analysis for the detected protein expression. **c** Sequence alignment of human miR-302 family miRNAs. **d** qRT-PCR indicates a significant down-regulation of miR-302 in MCF-7/ADR cells compared with MCF-7 cells. All graphs show means ± S.D. of three independent experiments

To better understand the biological mechanisms of chemoresistance in breast cancer cells and search for the reversion opportunities, we selected adriamycin sensitive and derived resistant breast cancer cell line pair(MCF-7 and MCF-7/ADR). To identify the differential sensitivity of the parental MCF-7 and MCF-7/ ADR breast cancer cell line to chemodrugs, we first determined the cytotoxicity of chemotherapeutic drugs, including adriamycin (ADR), paclitaxel(PAC) and etoposide(VP-16) by MTS assay, all of which are currently used for the treatment of breast cancer. As shown in Fig. 1a, MCF-7/ADR cells showed resistance to ADR, PAC and VP-16 with higher IC_50_ values (ADR:47.2 ± 4.33 μM, PAC:112.5 ± 10.16nM, VP-16: 1.072 ± 0.099 mM) than MCF-7 cells(ADR:1.02 ± 0.09 μM, PAC:3.57 ± 0.35nM, VP-16:75.7 ± 4.65 μM). The results showed that MCF-7/ADR cells had cross-resistance ADR, PAC and VP-16. We then characterized the differential expression of MDR-related ABC transporters, including MRP, P-gp, LRP, and BCRP, between the parental MCF-7 and its derivative ADR-resistant MCF-7/ADR breast cancer cells, using Western blot. As shown in Fig. 1b, the results showed that the MCF-7/ADR cell line displayed obviously increased levels of P-gp expression compared with the parental MCF-7 cell line, while the other three detected MDR proteins only showed slight upregulation in the MCF-7/ADR cell line. These data suggest that the overexpression of P-gp is one of the reasons that MCF-7/ ADR breast cancer cells are resistant to chemodrugs. Figure 1c shows that miR-302 members (302a, 302b, 302c, and 302d) share a high sequence homology, differing only in the 3′ hexanucleotides. We further examined the miR-302 in MCF-7 and MCF-7/ADR cells. qRT-PCR results showed that miR-302a, miR-302b, miR-302c and miR-302d were significantly downregulated in MCF-7/ADR cells compared with MCF-7 cells (Fig. 1d, *P* < 0.05).

### miR-302 overexpression sensitizes MCF-7 and MCF-7/ADR cells to ADR

**Fig. 2:**
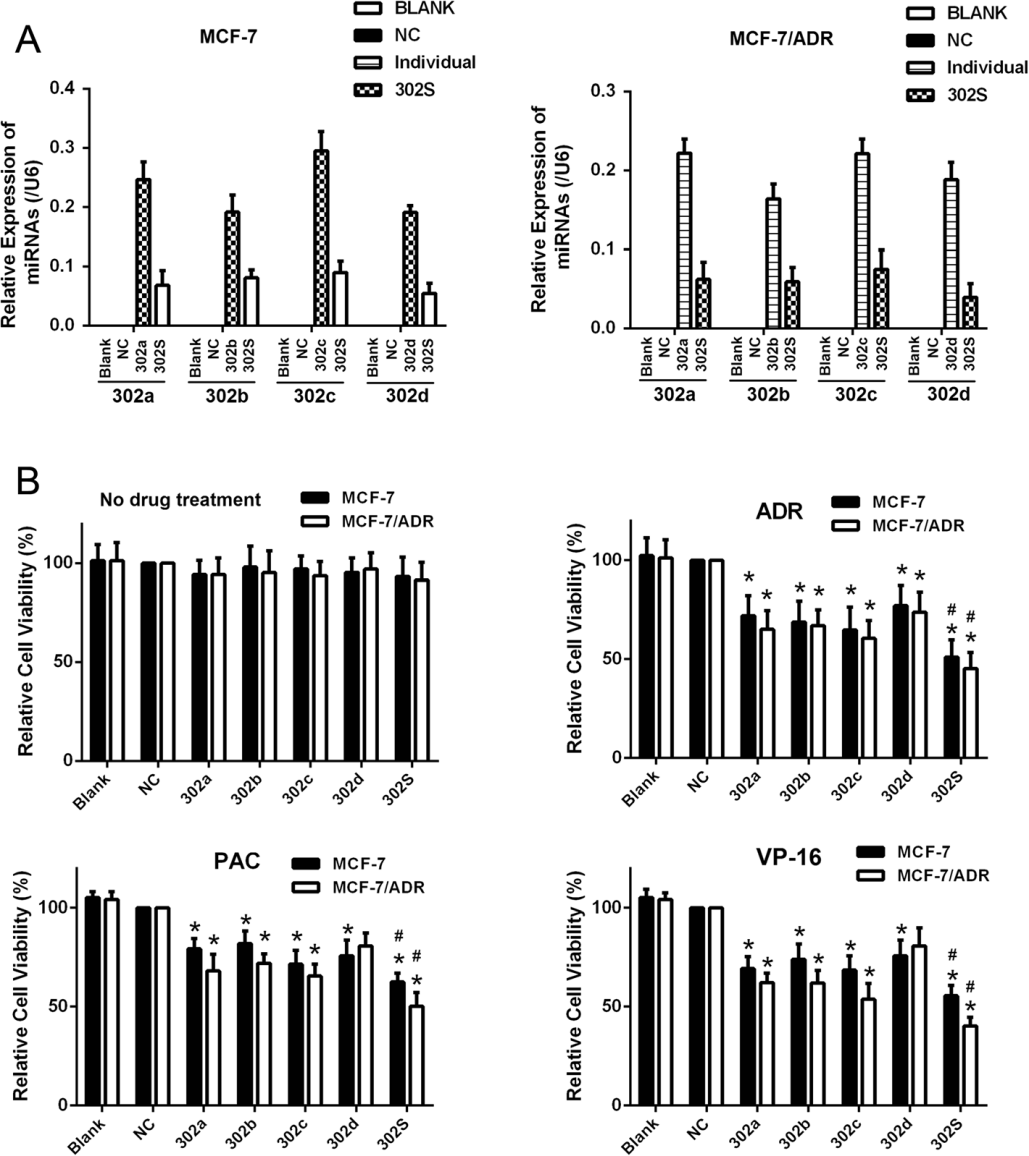
miR-302 modulated the resistance of human breast cancer cells to chemotherapeutic drugs. **a** qRT-PCR showing the relative expression of miR-302a/b/c/d in untreated MCF-7 and MCF-7/ADR cells (Blank), cells treated with miR-302a,b,c,d mimics (a final concentration of 20nM) or the four miRNAs mimic combination (miR-302S,the miRNAs mixed with miR-302a, miR-302b, miR-302c and miR-302d mimics by same concentration (4 × 5nM = 20nM) and negative controls (NC). **b** Following untransfection (Blank), transfection with either of the negative control RNA (NC) or miR-302 mimic, cells were treated with various concentrations of ADR, PAC and VP-16 for 48 h, respectively. The effect of the miR-302 mimic on the viability of MCF-7 cells and MCF-7/ADR in response to the three chemotherapeutic drugs was determined using the MTS assay. *n* = 3 (*P < 0.05 compared with the control group, ^#^ *P* < 0.05 compared with individual member)

To investigate the association of miR-302 expression with breast cancer chemoresistance, we determined the sensitivity of MCF-7 and MCF-7/ADR cells to ADR, PAC and VP-16 after treatment with miR-302a, miR-302b, miR-302c, miR-302d, and miR-302S mimics. The expression level of miR-302 was confirmed by qRT-PCR, showing that MCF-7 and MCF-7/ADR cells transfected with miR-302 mimics had higher miR-302 expression compared with those transfected with negative controls (Fig. 2a). miR-302 mimics alone had no significant effects on proliferation in MCF-7 and MCF-7/ADR cells (Fig. 2b). Following transfection of miR-302 mimics into MCF-7 and MCF-7/ADR, we treated the cells with a series of concentrations of ADR, PAC and VP-16. The inhibition of cell proliferation by the chemotherapeutic drugs was significantly increased by overexpression of miR-302a, miR-302b, miR-302d, and miR-302d in MCF-7 and MCF-7/ADR (Fig. 2b), suggesting that miR-302 sensitized MCF-7 and MCF-7/ADR cells to ADR, PAC and VP-16 cytotoxicity. Coexpression of miR-302a, miR-302b, miR-302c and miR-302d mimics combination (ie, four miRNA combination) had a stronger effect on reducing the cell growth compared with individual mimic (Fig. 2b).

### miR-302 increased ADR accumulation in MCF-7 and MCF-7/ADR cells

**Fig. 3:**
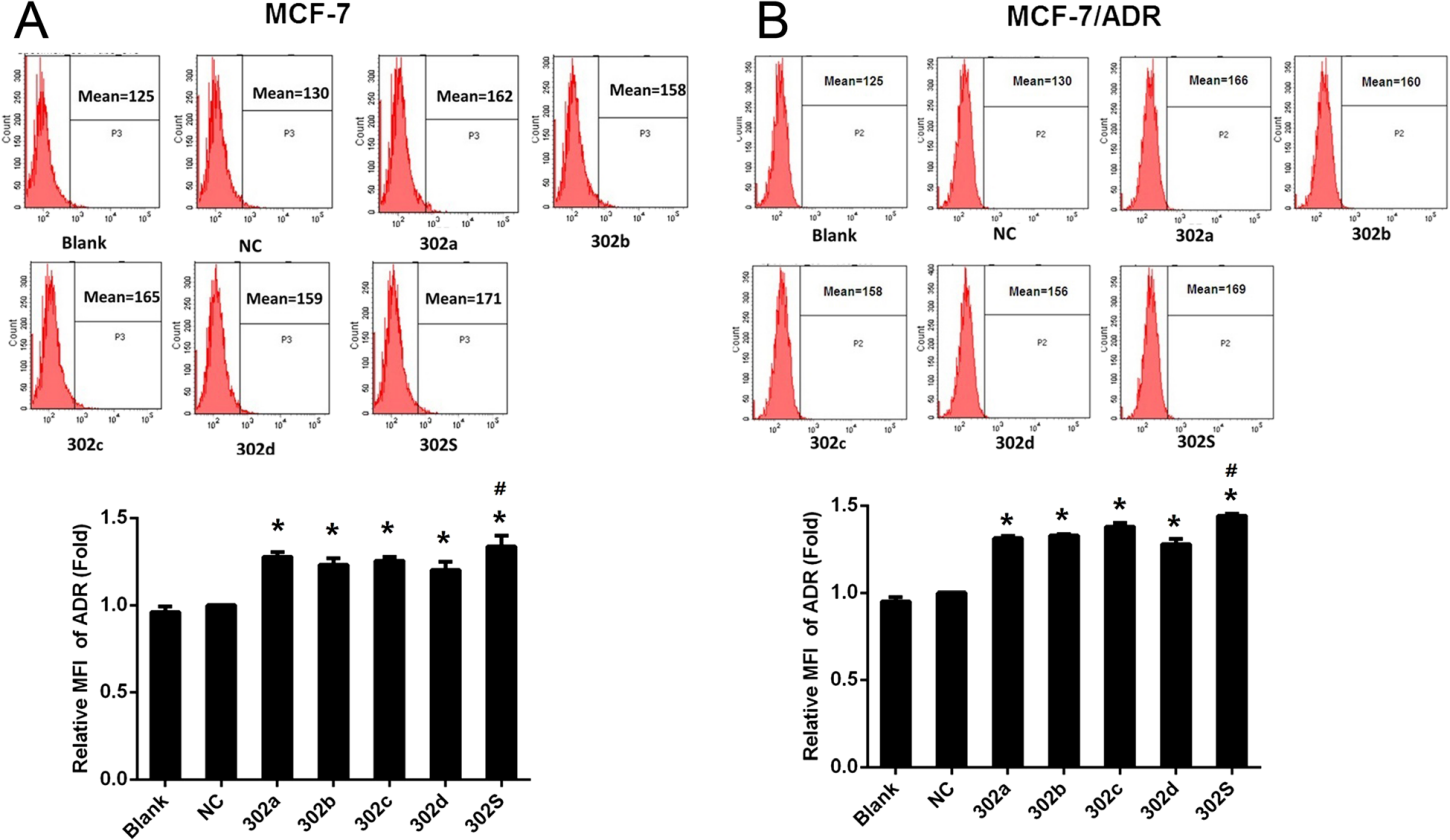
MiR-302 increased ADR accumulation in MCF-7 and MCF-7/ADR cells. Fluorescence intensity of ADR was detected by flow cytometry (up panel) and relative intensity of fluorescence (down panel) was analyzed at 48 h after transfection with miR-302 mimic or miR-NC followed by treatment with ADR at a final concentration of 1 μM for MCF-7 and 50 μM for MCF-7/ADR for 2 h in MCF-7 (**a**) and MCF-7/ADR (**b**) breast cancer cells.( *P < 0.05 vs NC. ^#^ *P* < 0.05 vs each individual member alone.) All graphs show means ± S.D. of three independent experiments

We also investigated the effect of miR-302a, miR-302b, miR-302c and miR-302d on ADR accumulation in MCF-7 and MCF-7/ADR cells. The intracellular ADR accumulation was evaluated after 2 h incubation with ADR. As shown in Fig. 3a and b, overexpression of miR-302a, miR-302b, miR-302c, and miR-302d significantly increased intracellular ADR accumulation in MCF-7 and MCF-7/ADR cells compared with controls. miR-302S treatment resulted in a significantly increase in intracellular ADR accumulation in the MCF-7 and MCF-7/ ADR cells than each individual member alone.

### MiR-302 inhibits P-gp expression more strongly than each individual member in MCF-7 and MCF-7/ADR cell

**Fig. 4:**
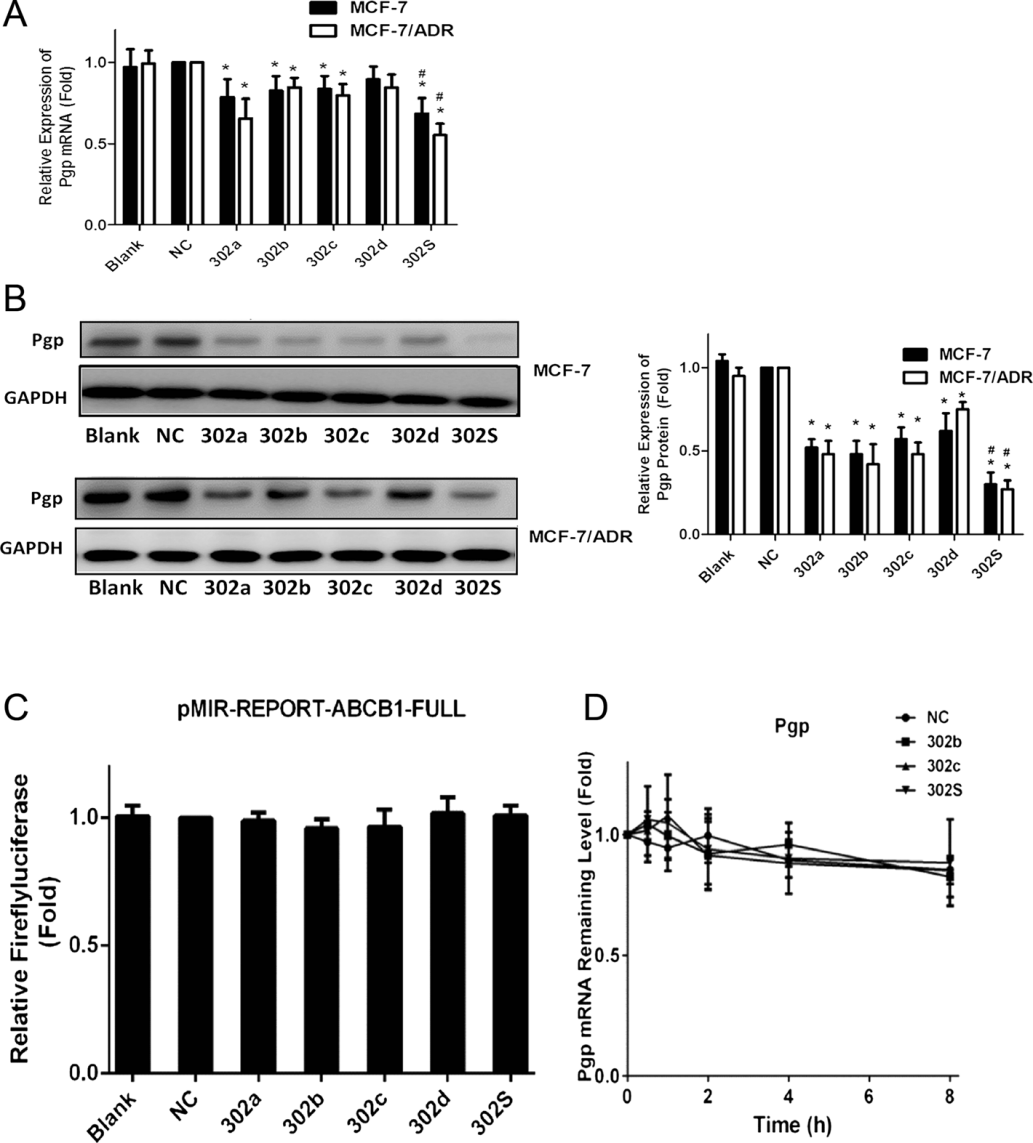
MiR-302 decreased P-gp mRNA and protein expression not through targeting the 3′-UTRs of P-gp in MCF-7 and MCF-7/ADR cells. **a** qRT-PCR showing the mRNA expression of P-gp in MCF-7and MCF-7/ADR cells. **b** Western blot and densitometric analysis showing the protein expression of P-gp in MCF-7 cell and MCF-7/ADR cell. **c** Dual-luciferase assays showing repression of wild-type UTR (ABCB1-3′UTR-FULL) following transfection of miR-302 and individual mimics or negative control mimic (NC). **d** The stability assay of P-gp mRNA. MCF-7/ADR cells were harvested at 0, 2, 4 and 6 h after treatment with 2 μg/ml actinomycin residual mRNAs was detected by qRT-PCR. All graphs show means ± S.D. of three independent experiments

Overexpression of the ATP-binding cassette transporter (ABC transporter) proteins P-gp in the cell membrane is known to promote active transport of ADR out of the cells. These processes decrease intracellular drug concentrations, and lead to multidrug resistance (MDR) of breast cancer cells. Therefore, expression levels of P-gp proteins are considered a useful clinical indicator of tumor cells’ drug sensitivity and patient prognosis [20]. Since we observed that miR-302 increased intracellular ADR accumulation, we sought to determine whether P-gp proteins were involved in the miR-302 overexpression-induced effects on MCF-7 and MCF-7/ADR cells. To investigate whether P-gp expression was regulated by miR-302, we transfected miRNAs mimics into MCF-7 and MCF-7/ADR cells and then evaluated P-gp expression levels. We found that the mRNA and protein expression of P-gp was significantly down-regulated in the miR-302a, miR-302b, miR-302c and miR-302d transfected cells compared with cells transfected with control miRNA. Further, combinations of four mimics had significant effects on the mRNA (Fig. 4a) and protein (Fig. 4b) level of P-gp compared with individual miRNA .

To investigate whether or not the 3′UTR of P-gp carries binding sites for the four miRNAs, we constructed a P-gp 3′UTR luciferase reporter vector, and co-transfected the four mimics individual or combination with P-gp mRNA 3′UTR reporter into HEK-293T cells. miR-302 had no significant effects on luciferase activity compared with control miRNA (Fig. 4c). We next tested whether or not the miRNA mimics regulate degradation of P-gp mRNA. We analyzed the stability of P-gp mRNA in MCF-7/ADR cells. Cells transfected with miR-302b or miR-302c mimic, were treated with vehicle or 5 μg/mL actinomycin D (to inhibit denovo RNA synthesis), and qRT-PCR were performed with primers amplifying P-gp mRNA. As shown in Fig. 4d, the half-life of P-gp mRNA in cells was not altered when miR-302 was overexpressed in MCF-7/ADR cells. These results suggest that miR-302b, miR-302c and miR-302S does not enhance the degradation of P-gp mRNA, implying that a mechanism other than direct mRNA degradation may be involved in miR-302 mediated P-gp inhibition.

### MiR-302 members directly target the 3′-UTR of MEKK1 and significantly suppress luciferase activity cooperatively

**Fig. 5:**
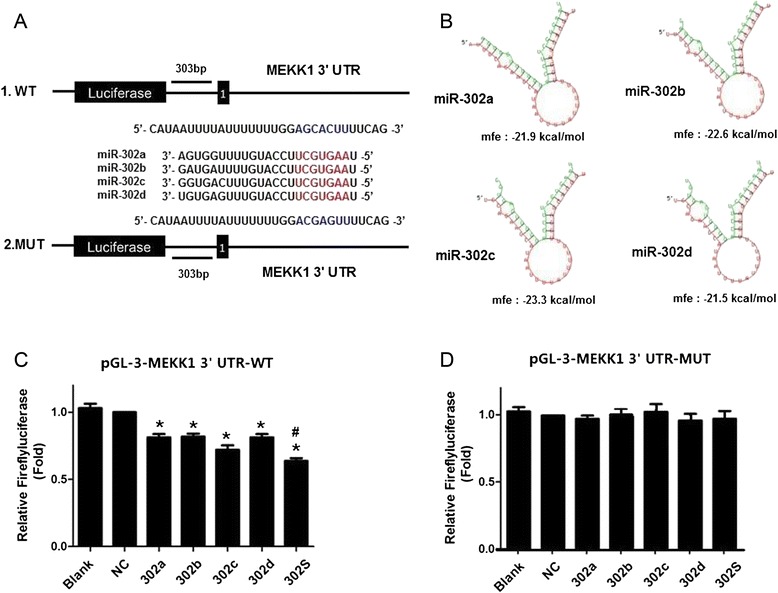
miR-302a,b,c and d directly targeted the 3′UTR of MEKK1 mRNA with cooperative effects. **a** Validation of the miR-302 target site in the 3′UTR of MEKK1 mRNA. The putative miR-302 targeted sequence in the MEKK1 mRNA. **b** RNAhybrid predicts one binding site of miR-302 in the MEKK1 3'-UTRs. The binding energy was calculated **c & d** Dual-luciferase assays showing repression of wild-type UTR MEKK1-3′UTR-FULL (**c**) or mutant UTR MEKK1-3′UTR-MUT (**d**) following transfection of miR-302 mimics or negative control mimic (NC). All graphs show means ± S.D. of three independent experiments. (* *P* < 0.05 vs. NC;^#^ *P* < 0.05 vs. Single)

Because our results showed that suppression of P-gp expression by miR-302a, miR-302b, miR-302c and miR-302d is not through typical miRNA-mediated mRNA degradation but at the level of protein and transcription, we hypothesized that a miR-302 target gene may function as a regulator of P-gp. To elucidate the mechanisms by which the miR-302a, miR-302b, miR-302c and miR-302d regulates P-gp expression, we used mRNA target-predicting algorithms (TargetScan, miRanda, and TargetRank) based on the presence of binding sites in the 3′UTR. Of these genes that overlapped among these algorithms, we found that MAP/ERK kinase kinase 1 (MEKK1), was one of the targets of miR-302a, miR-302b, miR-302c and miR-302d. Previous studies also have shown that MEKK1 phosphorylates the MAPK c-Jun amino-terminal kinase (JNK) [21], and activation of the JNK pathway can upregulate P-gp expression [22]. Therefore, in the next study, we choose MEKK1 as our target gene to study the mechanism of downregulated P-gp by miR-302. The 3′-UTRs of MEKK1 mRNA contained one putative miR-302 binding sites (Fig. 5a). The minimum free energy between miR-302 and the putative binding sites in the 3′-UTRs of MEKK1 mRNA suggested that miR-302 may target MEKK1 via binding these putative sites (Fig. 5b). To investigate whether MEKK1 is a direct target of miR-302, we generated a firefly luciferase reporter vector containing the MEKK1 3′UTR (Fig. 5a). The vector was transfected along with miRNA mimics into HEK-293T cells. Compared with the negative control, overexpression of each individual miRNAs significantly reduced luciferase activity by approximately 19 to 25 % (Fig. 5c). At the same dose, miR-302S inhibited luciferase activity by approximately 32 %, which was significantly more than each individual member alone (*P* < 0.05, Fig. 5c). Further, we generated mutant MEKK1 luciferase constructs, carrying base pair changes in miRNA putative binding site located at the MEKK1 3′UTR (Fig. 5a). All four miRNA mimics were transfected along with mutated MEKK1 vectors into HEK-293T cells to test the binding ability of different miRNA combinations. Mutation of the four miRNA-binding sequences reduced the ability of inhibiting luciferase activity by miR-302 (Fig. 5d), suggesting that they directly bind to the 3′UTR of MEKK1.

**Fig. 6:**
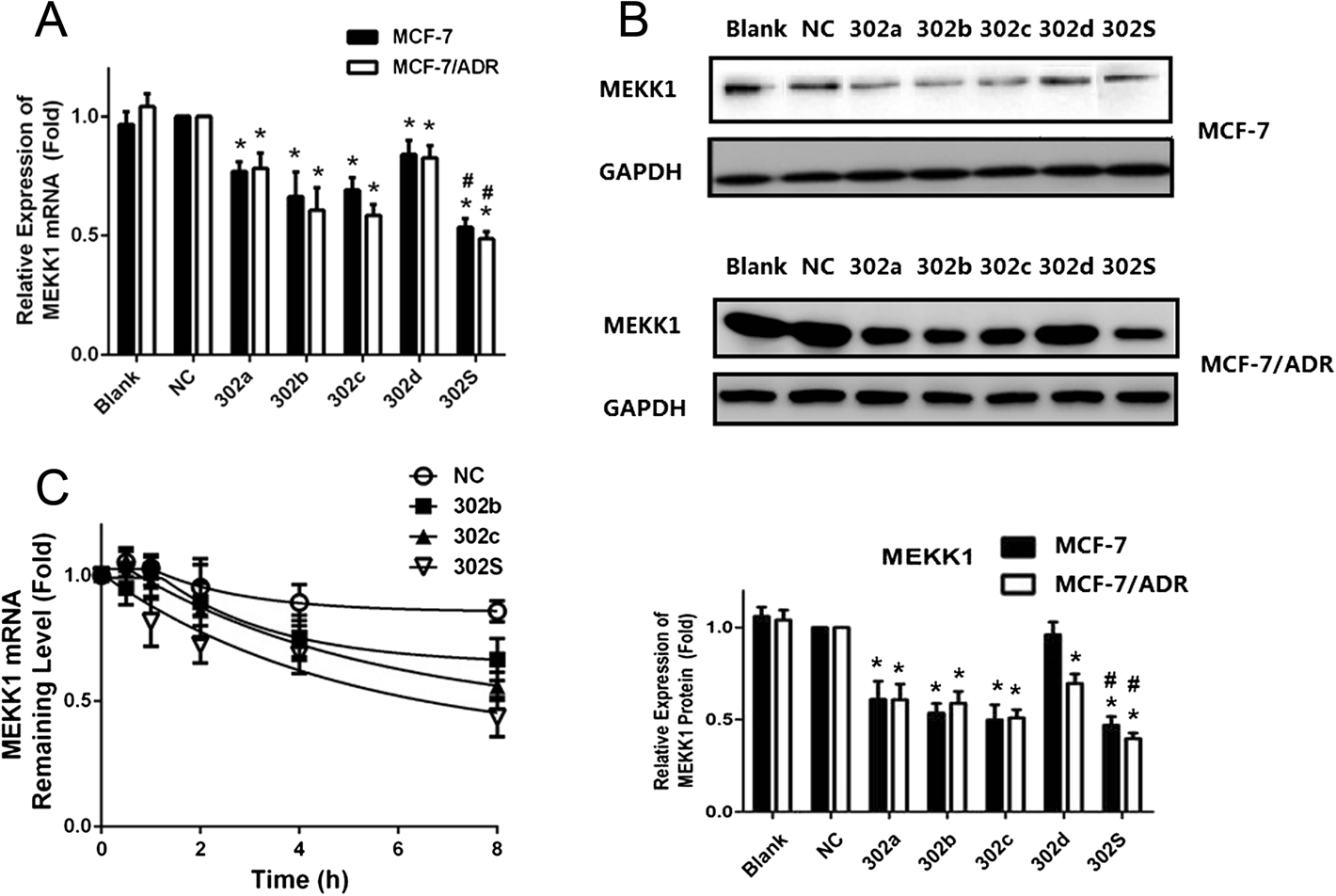
The relative expressions of MEKK1mRNA and protein in MCF-7 and MCF-7/ADR cells after miRNA transfection. **a** qRT-PCR showing the mRNA expression of MEKK1 in MCF-7and MCF-7/ADR cells. **b** Western blot and densitometric analysis showing the protein expression of MEKK1 in MCF-7 cell and MCF-7/ADR cell. **c** The stability assay of MEKK1 mRNA. MCF-7/ADR cells were harvested at 0, 2, 4, 6 and 8 h after treatment with 5 mg/mL actinomycin D. Residual mRNAs was detected by qRT-PCR. All graphs show means ± S.D. of three independent experiments. (* *P* < 0.05 vs. NC;^#^ *P* < 0.05 vs. Single)

We further studied the effect of miR-302 and each individual member on MEKK1 expression by overexpressing these miRNAs in MCF-7 and MCF-7/ADR cells. MEKK1 mRNA levels were confirmed by quantitative PCR analysis 48 h post-transfection. A significant decrease in MEKK1 mRNA levels was found in cells transfected with each individual miRNAs, compared with controls (miR-NC). In contrast, when the four miRNAs were transfected together, we observed a more significant decrease in MEKK1mRNA levels (*P* < 0.05, Fig. 6a), which is consistent with the findings of the luciferase experiments described above. Similarly, a 50 % reduction in MEKK1 protein levels was observed when the four miRNAs were transfected together, suggesting again that they act cooperatively (Fig. 6b).

RNA degradation experiments were conducted to assess if miR-302 affect MEKK1 mRNA stability. The data showed that miR-302b and miR-302c accelerated the decay of MEKK1 mRNA, compared with the control. The higher rate of mRNA decay caused by miR-302S than individual was also found (Fig. 6c). The results suggest that mRNA degradation could be an important mechanism underlying miR-302-mediated posttranscriptional regulation of MEKK1.

### MiR-302 decreases MEKK1-mediated ERK MAP kinase signaling pathway in MCF-7 and MCF-7/ADR cells

**Fig. 7:**
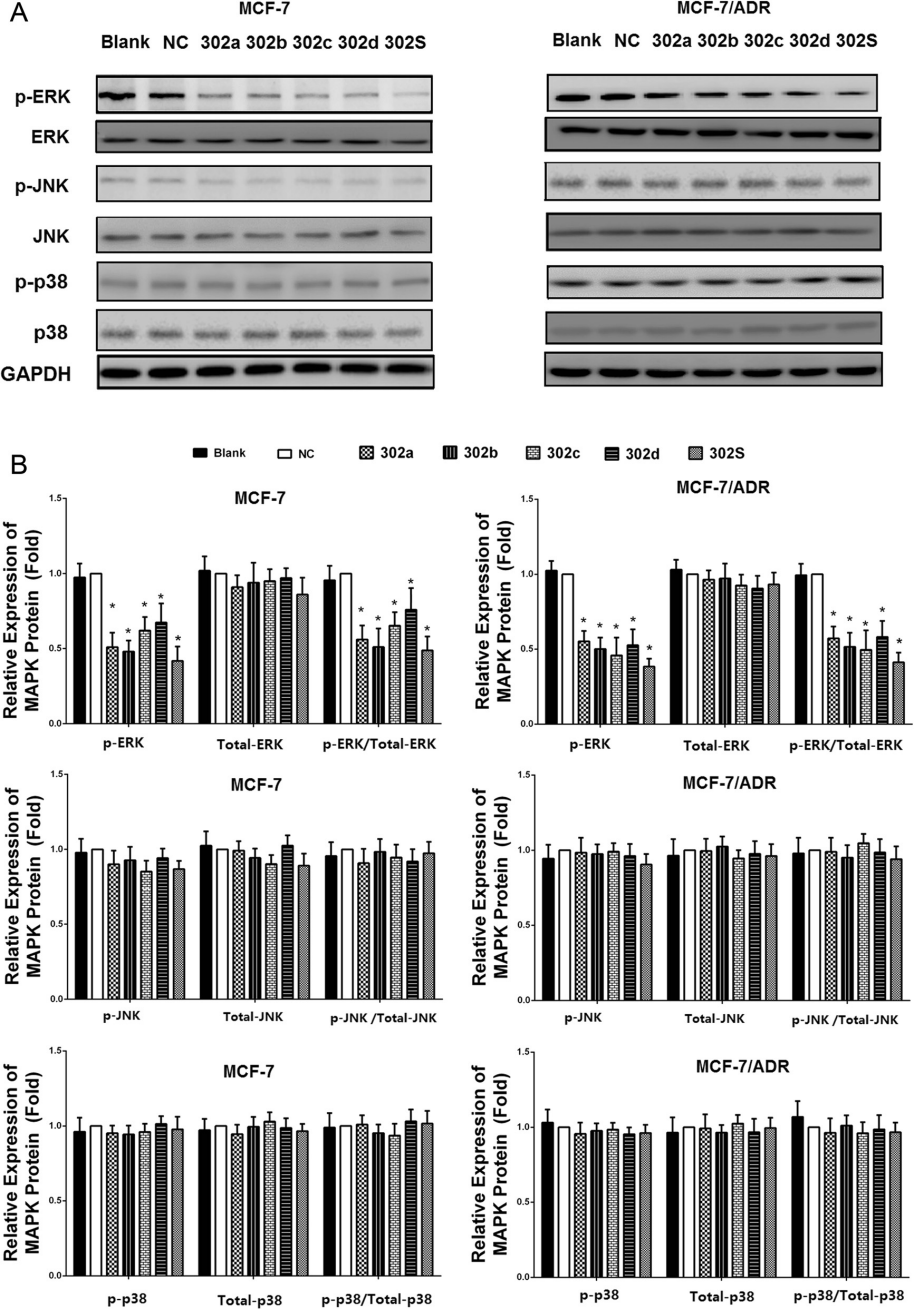
Overexpression of miR-302 decreased the expression of the ERK signal pathway. **a** MCF-7 and MCF-7/ADR cells were transfected with miR-302a, miR-302b, miR-302c, miR-302d, miR-302S mimic, respectively. Total cellular proteins (50 μg) from exponentially growing cells treated as indicated in the figure were subjected to western blot analysis with antibodies directed against the proteins or their phosphorylated form as indicated. GAPDH was applied as control for equal loading. All experiments were carried three times independently. **b** Densitometric analysis for the detected protein expression

We found that miR-302 negatively regulates MEKK1 by targeting the MEKK1 mRNA-3′UTR in MCF-7 and MCF-7/ADR cells. In addition, MEKK1 can phosphorylate and activate ERK and JNK MAPK pathway. We then sought to determine whether the MEKK1-mediated downstream signal pathway was also impacted by miR-302. Therefore, we examined changes in the expression of proteins association with the MAPK pathway, including p38, JNK and ERK in breast cancer cells following miR-302 overexpression. The results showed that after transfection with miR-302 mimics in MCF-7 and MCF-7/ADR cells, the expression of phosphorylation levels of ERK was decreased (Fig. 7a and b). Moreover, the four miRNAs combination effects were notably higher than those treated with individual miRNA. The total and phosphorylated protein levels of JNK and p38 were unaffected by miR-302 mimics transfection in any of the cell types (Fig. 7a and b). These results indicated that miR-302 may be an important regulator of ERK signaling pathway.

### MEKK1-mediated ERK pathway activation accounts for the decreased P-gp expression induced by overexpression of miR-302

**Fig. 8:**
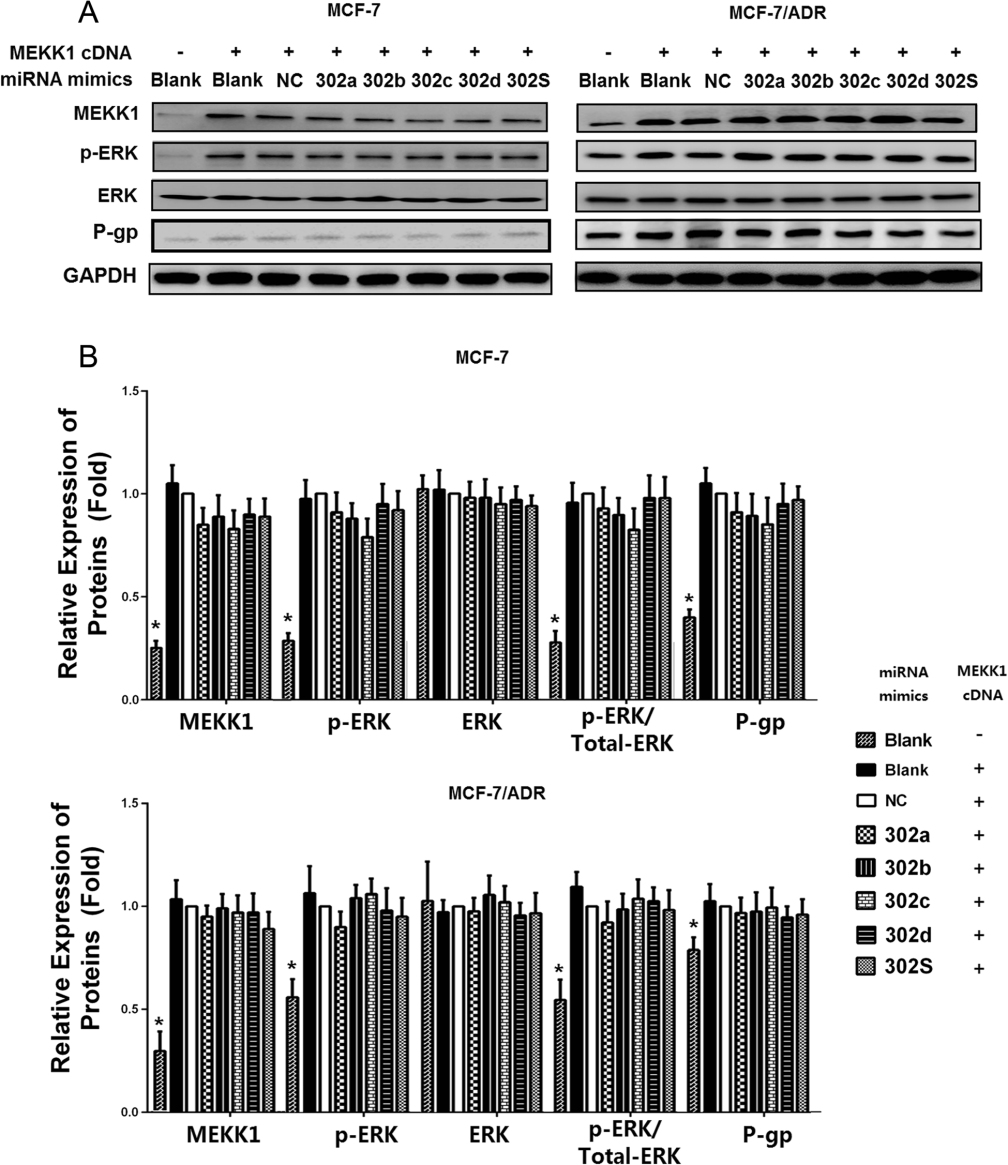
Overexpression of MEKK1 restored the inhibition of P-gP induced by miR-302. **a** Cells were co-transfected with miRNA mimics indicated in the figure and MEKK1- cDNA(lacking the 3′UTR sequence ) for 48 h, then lysates were harvested and subjected to immunoblotting with anti-MEKK1, anti-ERK, anti-p-ERK, and anti-P-gP antibodies. GAPDH was applied as control for equal loading. All experiments were carried three times independently. **b** Densitometric analysis for the detected protein expression. (* *P* < 0.05 vs. NC)

In the previous experiments, we found that induced expression of miR-302 led to an increased drug sensitivity. These changes appeared to be due to inhibition of P-gp expression. ERK signaling pathway is known to cause activation and expression of P-gp [23]. We also found that miR-302 suppressed ERK pathway in breast cancer cells. To further investigate whether inhibition of MEKK1-mediated ERK pathways was involved in the miR-302 induced P-gp suppression, MCF-7 and MCF-7/ADR cells were co-transfected miR-302 mimics with MEKK1 cDNA(lacking the 3′UTR sequence) inducing expression of MEKK1. The results showed that co-transfection of pcDNA3.1(+)-MEKK1 and miR-302 mimics increased MEKK1 expression (Fig. 8a and b). miR-302 suppressed P-gp suppression and ERK pathway, which was reversed by MEKK1 overexpression (Fig. 8a and b). These results demonstrated that P-gp suppression by miR-302 is mediated by down-regulation of MEKK1-mediated ERK pathway. Collectively, these results indicate that the synergistic overexpression of these four miRNAs sensitizes breast cancer cells to ADR more effectively than individual miRNA alone. This might be due in part to the indirectly suppressing P-gp by targeting MEKK1-mediated ERK pathway.

## Discussions

Although chemotherapy has greatly improved the prognosis of breast cancer, drug-resistance still remains as the main obstacle of successful treatment. Recently, miRNAs were reported to be differentially expressed in drug resistant cancers and could regulate the drug resistance. In the present study, we found that miR-302 cluster were differentially expressed between MCF-7/ADR and MCF-7. In the transfection experiments, we found alteration of miR-302 expression could change the degree of ADR, PAC and VP-16 resistance in MCF-7 and MCF-7/ADR. Previous studies showed that miR-302 cluster are highly expressed in embryonic stem cells (ESCs) and induced pluripotent stem cells (iPSCs), and their expression levels rapidly decline as pluripotent stem cells begin to differentiate [24]. Recent studies have shown that miR-302 targets epigenetic regulators (AOF1/2, MECP1-p66, MECP2 and MBD2) [25], cell-cycle regulators (Cyclin D1/D2, CDK2, BMI-1 and PTEN [26]), TGF-β regulators (Lefty1/2 [27] and TGFBR2 [28]), BMP inhibitors (DAZAP2, SLAIN1, and TOB2) [29] and NR2F2 [30]. Most studies about the role of miR-302 have been done in ESCs, but the function of miR-302 in cancer has not been studied. Currently, a few studies focused on the role of miR-302 in tumor, Yang CM et al. found that expression of the miR-302/367 cluster in glioblastoma cells suppresses tumorigenic gene expression patterns and abolishes transformation related phenotypes [31]. The results of the current study provide the evidence to suggest that miR-302 play an important role in drug sensitivity.

Furthermore, we also found that ectopic the miR-302a/b/c/d combination expression sensitizes MCF-7/ADR cells to ADR more efficiently than inducing an individual miRNA. This effect cannot be attributed to the miRNA dose, since the same amount of total miRNA was used irrespective of whether they were transfected alone or in combination. We show that the combined overexpression of miRNAs (miR-302a, miR-302b, miR-302c and miR-302d) could potentially be used as a therapy for breast cancer. However, additional animal and human studies are warranted. Based this results, we highlighted the importance of a combination of miRNAs as a therapeutic target, a concept that could be used successfully to promote sensitivity in other cancers.

Our study found the expression of P-gp levels in the MCF-7/ADR was significantly higher than that in the MCF-7, suggesting that over-expression of P-gp may be one of mechanism of drug resistance formation in MCF-7/ADR cells. P-gp is an ATP-dependent membrane pump that transports the drug out of cells, resulting in multidrug resistance. Therefore, inhibition of P-gp transporter function or inhibition of its expression may reverse the MDR phenotype through enhancing intracellular accumulation of anticancer drugs. There has been a worldwide effort investigating chemical agents for their ability to overcome MDR through interacting with P-gp and inhibiting its transporter function. These MDR modulators include calcium channel blockers [32], calmodulin inhibitors [33], and other classes of compounds [34]. Recently, several miRNAs have been identified as critical regulators of P-gp mediated drug resistance in many cancer. For instance, miR-451 overcame the doxorubicin resistance by downregulating P-gp expression in the doxorubicin resistant breast cancer cell lines MCF-7/ADR [35]. Inhibition of miR-27a enhanced the paclitaxel sensitivity in A2780/Taxol by modulating MDR1/P-gp expression [36]. Inhibiting the expression of miR-130a resulted in down-regulating of MDR1 mRNA and P-gp in A2780/DDP ovarian cancer cell line [13]. In this study, we proved that overexpression of miR-302 resulted in down-regulating of MDR1 mRNA and P-gp. ADR accumulation was also increased in miR-302 mimic-transfected MCF-7 and MCF-7/ADR cells. These results indicate that miR-302 reduces the efflux of cytotoxic drugs by downregulating the expression of the ABC transporter P-gp.

However, bioinformatics analysis and luciferase reporter assay experiment showed no direct binding site of miR-302 in MDR1 mRNA-3′UTR, How might P-gp expression be down-regulated by elevated miR-302? Here, we identified MEKK1 as the functional target, through which miR-302 regulates P-gp expression and chemoresistance. The interaction of miR-302 with the 3′UTR of the MEKK1 mRNA was confirmed by the luciferase assay, in which the luciferase activity of the reporter gene harboring 3′UTR of MEKK1 mRNA was significantly inhibited by miR-302. Furthermore, we found that miR-302a, miR-302b, miR-302c and miR-302d mimics inhibited the expression of MEKK1 in MCF-7 and MCF-7/ADR cells, further suggesting that miR-302 specifically down-regulated MEKK1. To directly demonstrate that suppression of MEKK1 by miR-302 was responsible for the decreased P-gp expression, we performed a rescue experiment by expressing MEKK1 cDNA in MCF-7 and MCF-7/ADR cells. Strikingly, expression of the MEKK1 rescued the down-regulatory effect of miR-302 on P-gp in MCF-7/ADR cells. Thus, these results indicated a crucial requirement for MEKK1 in mediating miR-302-induced down-regulation of P-gp.

MEKK1 is an upstream activator of p38, JNK and ERK1/2 through its kinase domain [37]. Previous studies have shown that activation of p38,JNK and ERK cooperatively increase transport activity of P-gp and induce a multidrug resistant phenotype [38], we showed in this study that miR-302 decreased ERK activation in the MCF-7 and MCF-7/ADR cells. It is therefore very likely that miR-302 inhibited MEKK1 expression via binding to the 3′ UTR of MEKK1 mRNA, which in turn decreased ERK activation, thereby leading to the repression of MDR1 mRNA expression and ultimately the repression of P-gp protein expression.

Several studies have shown that different miRNAs can cooperatively regulate the same target gene. For example, Georges et al. reported that miR-192 and miR-215 cooperatively regulated cell cycle transcripts [39]. Frampton et al. showed that miR-21, miR-23a, and miR-27a cooperatively inhibited tumor suppressor genes to promote pancreatic tumor growth and progression. Kotani et al. found that miR-128b and miR-221 cooperatively sensitized MLL-AF4 acute lymphocytic leukemia cells to glucocorticoids [40]. Our study showed that miR-302S exhibited more strong effects on inhibition of P-gp and MEKK1 expression than each individual member alone, suggesting that miR-302 members cooperatively inhibited P-gp and MEKK1 expression. Several studies have shown that miRNAs reduce protein expression predominantly by destabilization of the target mRNA. In the present study, we found that miR-302S accelerated the decay of MEKK1 mRNA compared with miR-302b and miR-302c, suggesting that miR-302 members may cooperatively promote degradation of MEKK1 mRNA.

## Conclusions

The results of this study demonstrate that miR-302 increases the sensitivity of breast cancer cells to the anticancer agent ADR, PAC and VP-16 by downregulating P-gp expression. Most importantly, we found that miR-302S produced stronger effects than each individual member alone, suggesting that miR-302 members may cooperatively downregulate P-gp expression to increase chemosensitivity of breast cancer cells. Additionally, MEKK1 was confirmed as a functional target of miR-302 in breast cancer cells, miR-302 sensitizes breast cancer cells to chemodrugs via suppressing P-gp by targeting MEKK1 of ERK pathway. These results enhance our understanding of the molecular network underlying MDR in breast cancer.
