# Correction to: MiR-302a/b/c/d cooperatively sensitizes breast cancer cells to adriamycin via suppressing P-glycoprotein(P-gp) by targeting MAP/ERK kinase kinase 1 (MEKK1)

**DOI:** 10.1186/s13046-022-02312-6

**Published:** 2022-03-12

**Authors:** Lin Zhao, Yan Wang, Longyang Jiang, Miao He, Xuefeng Bai, Lifeng Yu, Minjie Wei

**Affiliations:** Department of Pharmacology, School of Pharmacy, China Medical University, No.77 Puhe Road, Shenyang North New Area, Shenyang City, 110122 Liaoning China

**Correction to: J Exp Clin Cancer Res 35, 25 (2016)**

**https://doi.org/10.1186/s13046-016-0300-8**

Following publication of the original article [1], the authors identified minor errors in Figs. 6 and 7; specifically:

- Fig. 6b: incorrect band used for MEKK1 band of MCF-7 cells; correct band is now used
- Fig. 7a: incorrect band used for p38 band of MCF-7 cells; correct band is now used

The corrected figures are given here. The correction does not have any effect on the final conclusions of the paper.

**Fig. 6:**
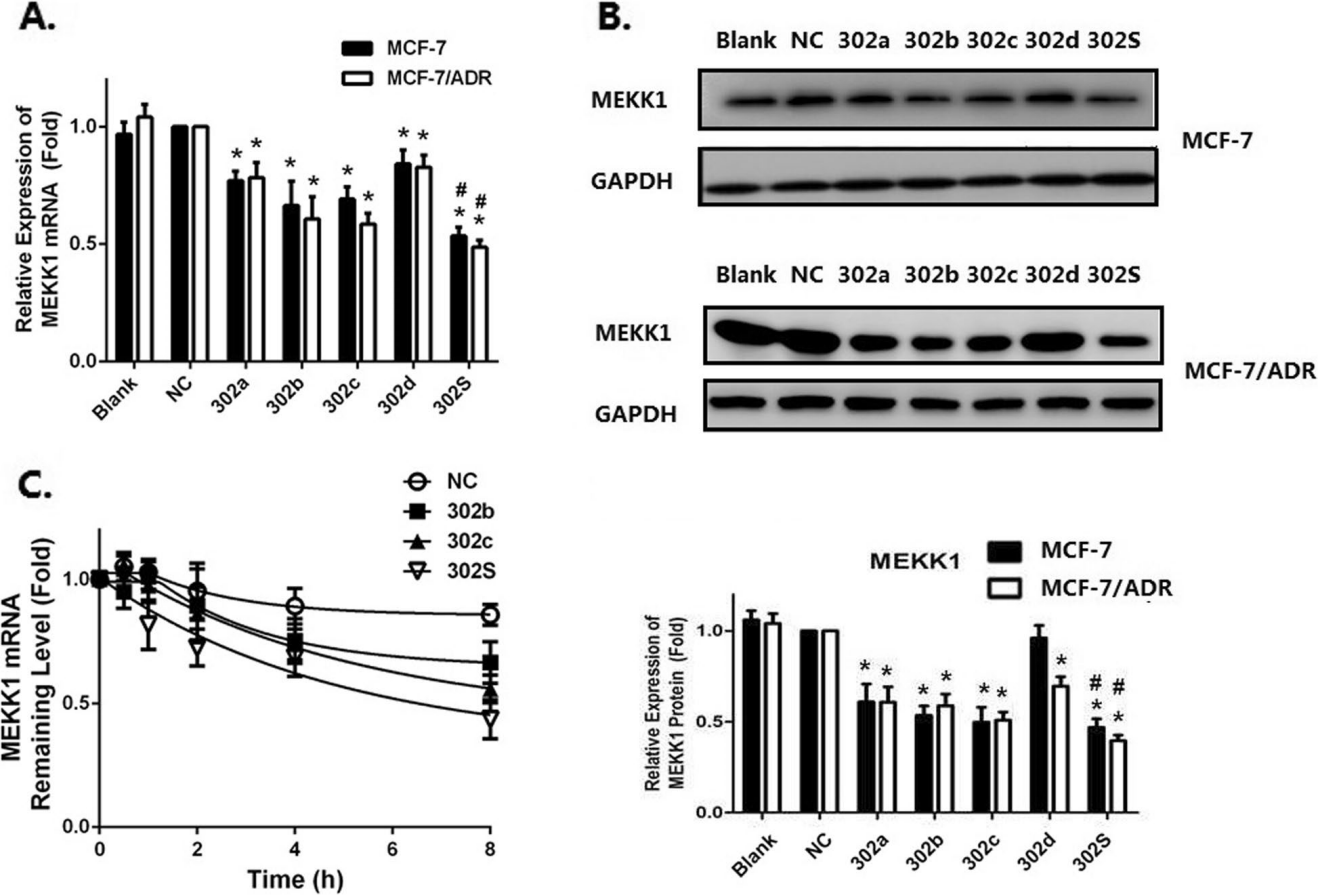
The relative expressions of MEKK1mRNA and protein in MCF-7 and MCF-7/ADR cells after miRNA transfection. **a** qRT-PCR showing the mRNA expression of MEKK1 in MCF-7and MCF-7/ADR cells. **b** Western blot and densitometric analysis showing the protein expression of MEKK1 in MCF-7 cell and MCF-7/ADR cell. **c** The stability assay of MEKK1 mRNA. MCF-7/ADR cells were harvested at 0, 2, 4, 6 and 8 h after treatment with 5 mg/mL actinomycin D. Residual mRNAs was detected by qRT-PCR. All graphs show means ± S.D. of three independent experiments. (^*^P < 0.05 vs. NC;^#^*P* < 0.05 vs. Single)

**Fig. 7:**
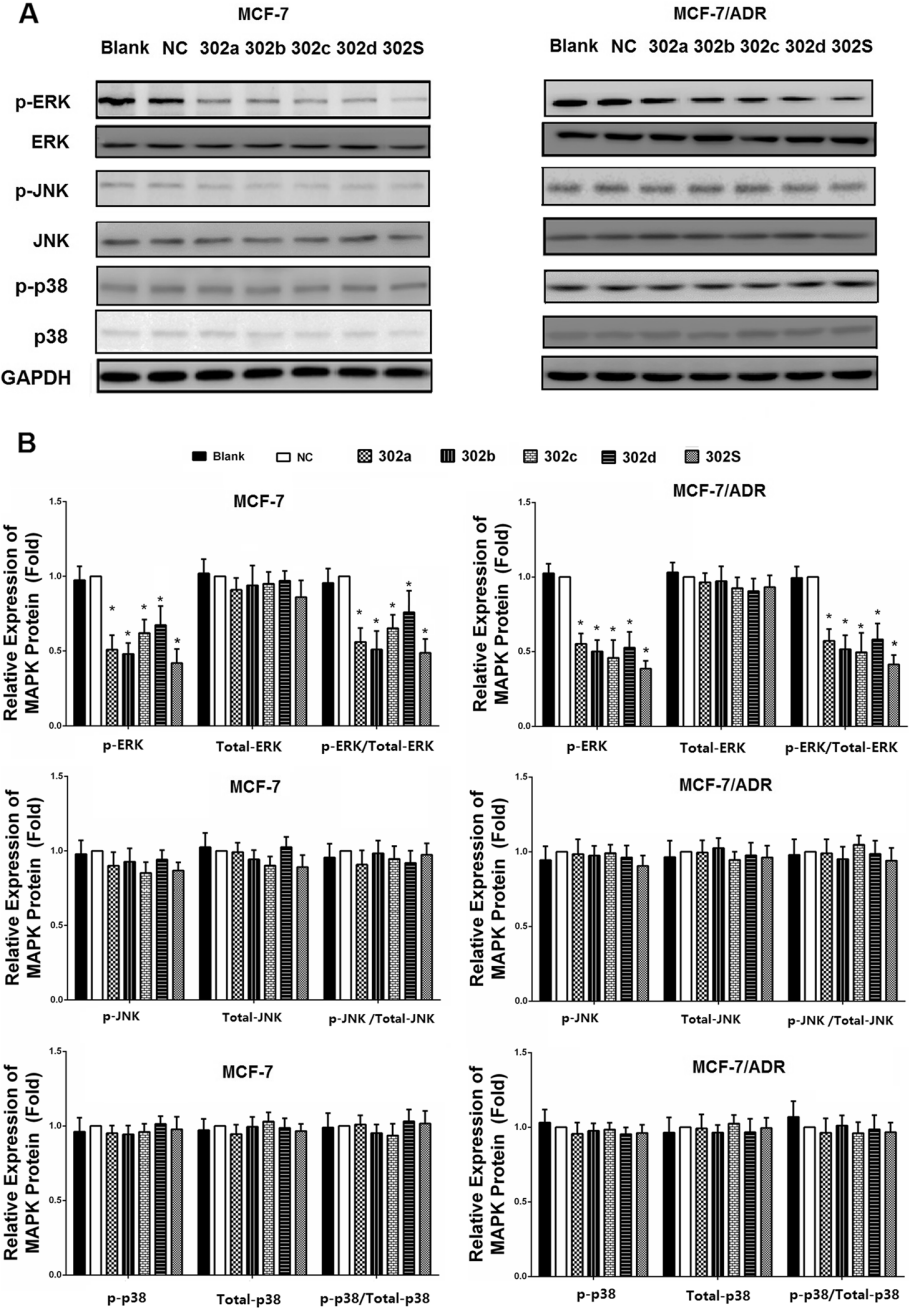
Overexpression of miR-302 decreased the expression of the ERK signal pathway. **a** MCF-7 and MCF-7/ADR cells were transfected with miR-302a, miR-302b, miR-302c, miR-302d, miR-302S mimic, respectively. Total cellular proteins (50 μg) from exponentially growing cells treated as indicated in the figure were subjected to western blot analysis with antibodies directed against the proteins or their phosphorylated form as indicated. GAPDH was applied as control for equal loading. All experiments were carried three times independently. **b** Densitometric analysis for the detected protein expression
